# Genomic Analysis of CTX-M-Group-1-Producing Extraintestinal Pathogenic *E. coli* (ExPEc) from Patients with Urinary Tract Infections (UTI) from Colombia

**DOI:** 10.3390/antibiotics9120899

**Published:** 2020-12-13

**Authors:** Elsa De La Cadena, María Fernanda Mojica, Nathaly Castillo, Adriana Correa, Tobias Manuel Appel, Juan Carlos García-Betancur, Christian José Pallares, María Virginia Villegas

**Affiliations:** 1 Grupo de Investigación en Resistencia Antimicrobiana y Epidemiologia Hospitalaria, Universidad El Bosque, Bogotá 110121, Colombia; mfm72@case.edu (M.F.M.); ncastillop@unbosque.edu.co (N.C.); tobiasm.appel@gmail.com (T.M.A.); jgarciab@unbosque.edu.co (J.C.G.-B.); icako@hotmail.com (C.J.P.); mariavirginia.villegas@gmail.com (M.V.V.); 2 Department of Infectious Diseases, Case Western Reserve University, Cleveland, OH 44106-7164, USA; 3 Research Service, Louis Stokes Veterans Affairs Medical Center, Cleveland, OH 44106-7164, USA; 4 Centro Internacional de Entrenamiento e Investigaciones Médicas (CIDEIM), Cali 760031, Colombia; adriana.correa@imbanaco.com.co; 5 Facultad de Ciencias Básicas, Universidad Santiago de Cali, Cali 760031, Colombia; 6 Comité de Infecciones y Vigilancia Epidemiológica, Centro Médico Imbanaco, Cali 760031, Colombia

**Keywords:** *E. coli* ST131, O25b:H4, H30-RX subclone, CTX-M, urinary tract infections (UTI), clonal dissemination

## Abstract

Background: The dissemination of the uropathogenic O25b-ST131 *Escherichia coli* clone constitutes a threat to public health. We aimed to determine the circulation of *E. coli* strains belonging to O25b:H4-B2-ST131 and the *H*30-Rx epidemic subclone causing hospital and community-acquired urinary tract infections (UTI) in Colombia. Methods: Twenty-six nonduplicate, CTX-M group-1-producing isolates causing UTI in the hospital and community were selected for this study. Results: Twenty-two *E. coli* isolates harboring CTX-M-15, one CTX-M-3, and three CTX-M-55 were identified. Multilocus Sequence Typing (MLST) showed a variety of sequence types (STs), among which, ST131, ST405, and ST648 were reported as epidemic clones. All the *E. coli* ST131 sequences carried CTX-M-15, from which 80% belonged to the O25b:H4-B2 and *H*30-Rx pandemic subclones and were associated with virulence factors *iss*, *iha*, and *sat*. *E. coli* isolates (23/26) were resistant to ciprofloxacin and associated with amino acid substitutions in quinolone resistance-determining regions (QRDR). We detected two carbapenem-resistant *E. coli* isolates, one coproducing CTX-M-15, KPC-2, and NDM-1 while the other presented mutations in *ompC*. Additionally, one isolate harbored the gene *mcr-1*. Conclusions: Our study revealed the circulation of the *E. coli* ST131, O25b:H4-B2-*H*30-Rx subclone, harboring CTX-M-15, QRDR mutations, and other resistant genes. The association of the *H*30-Rx subclone with sepsis and rapid dissemination warrants attention from the public health and infections control.

## 1. Introduction

The spread of antimicrobial-resistant microorganisms has become a major threat to global public health, as infections caused by such bacteria are often responsible for the increase in patient mortality and morbidity due to inappropriate therapy [1]. Extended spectrum β-lactamases (ESBLs) are enzymes that provide resistance to penicillins; oxyimino-cephalosporins (like ceftazidime, ceftriaxone, and cefotaxime); and monobactams but not to cephamycins (cefoxitin) or carbapenems [2]. The acquisition of ESBLs is the main mechanism of resistance to oxyimino-cephalosporins in *Escherichia coli*, which are a major cause of hospital- and community-acquired urinary tract infections (UTI) [3]. The overall prevalence of ESBLs has increased globally, mainly due to the spread of CTX-M enzymes. To date, more than 220 different CTX-M types have been recorded, CTX-M-15 being the most widely distributed [4]. Global surveillance studies have shown high rates of ESBL-producing *E. coli* in certain areas of Asia, Africa, and Latin America, which has led to an increased use of last-resort antimicrobial drugs such as carbapenems, leading to the emergence of carbapenem-resistant bacteria [5].

The population structure of ESBL-producing *E. coli* is dominated globally by a high-risk clone named sequence type (ST) 131, which is associated with fluoroquinolone and extended-spectrum cephalosporin resistance and has played a critical role in the current pandemic spread [6]. ST131 has been grouped into three clades, which are associated with specific *fimH* alleles: A clade (*fimH*41-O16), B clade (*fimH*22-O25b), and C clade (*fimH*30-O25b, including *fimH*30-Rx). Whole-genome sequencing (WGS) studies identified two subclades within clade C (*H*30-R and H30-Rx). The most prevalent lineage within ST131 is *fimH*30, a *H*30 variant of the type 1 fimbrial adhesin gene, and its subclones *H*30-R and *H*30-Rx [7]. Of special interest, isolates belonging to the *H*30-Rx subclone of ST131 have been associated with upper urinary tract infections (UTIs) and primary sepsis [7].

Other important epidemic clones detected among ESBL producers include ST405, ST38, ST648, ST410, and ST1193 [8]. In 2011, the presence of CTX-M-15-producing ST131 and ST405 *E. coli* isolates was reported for the first time in Colombia [9]. However, the circulation of the *E. coli* ST131 clone, B2, O25b:H4, and the *H*30-Rx subclone of extraintestinal pathogenic *E. coli* (ExPEC) in the country remains unexplored. Herein, we determined the circulation of CTX-M-15, O25b:H4, and the *H*30-Rx subclone in ExPEC from hospital or community origins, causing UTI and blood stream infections (BSIs) in Colombia.

## 2. Results

### 2.1. Antibiotic Susceptibility

**Figure 1:**
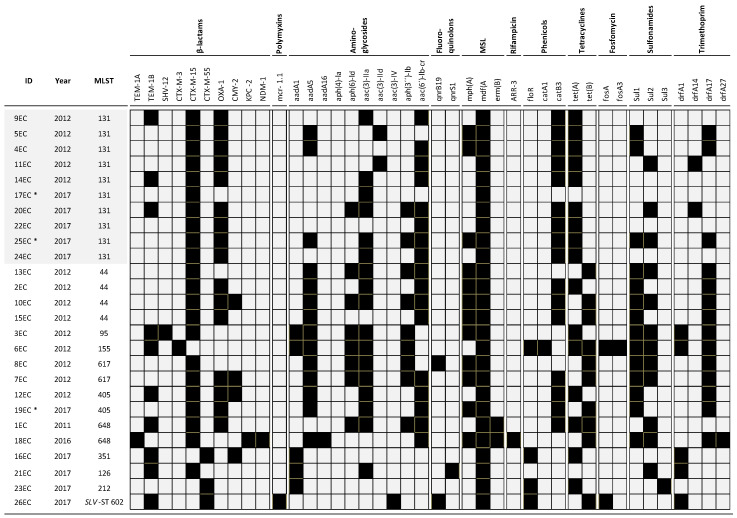
Antibiotic resistance genes found in the CTX-M *Escherichia coli* strains. Black squares represent the presence of the respective antibiotic resistance gene, and grey represents its absence. (*) Isolates recovered from blood samples. MLST: Multilocus Sequence Typing.

Antimicrobial susceptibilities of all strains evaluated are shown in Table 1. As expected, all isolates were resistant to cefotaxime and ceftriaxone; a resistance to ceftazidime was found in 21/26 (81%) of the isolates, to cefepime in 18/26 (69%), to piperacillin-tazobactam in 2/26 (8%), and to fosfomycin in 1/26 (4%) of the isolates. In addition, 92% susceptibility to ertapenem was found. The complete resistome of the isolates is presented in Figure 1.

### 2.2. Molecular Characterization of β-Lactamase Genes

As shown, the occurrence of ESBL genes remained as follows: *bla*_CTX-M-15_ in 22/26 (85%) isolates, *bla*_CTX-M-55_ in three (12%) isolates, *bla*_CTX-M-3_, and *bla*_SHV-12_ with one isolate each (4%) (Figure 1). The prevalence of other β-lactamase-encoding genes, such as *bla*_TEM-1,_ *bla*_OXA-1_, and *bla*_CMY-2_ were found in 38%, 54%, and 11% of the evaluated isolates, respectively. Notably, the acquired resistance genes identified among ST131 isolates included *bla*_OXA-1_, *aac-(6’)lb-cr*, *mdfA*, *tetA*, and *catB3*. We detected two carbapenem-resistant isolates (18EC and 23EC isolates), one of them coproducing CTX-M-15, KPC-2, and NDM-1 and the other with point mutations, insertions, and deletions in *ompC*. Additionally, one isolate harbored the resistance gene *mcr-1*. None of these three isolates belonged to ST131.

### 2.3. Multilocus Sequence Typing (MLST) and Phylogenetic Analysis

The molecular typing information of the isolates is presented in Table 2. According to the MLST protocol applied, the 26 *E. coli* isolates were distributed into 11 different STs, including a new ST, a single locus variant of ST602. The most common ST was ST131 (10/26 isolates; 38%), followed by ST44 (4/26 isolates; 15%) and ST405, ST617, and ST648 (2/26 isolates; 8% each). Lastly, ST95, ST155, ST351, ST126, ST212, and *SLV*-ST602, with one isolate each. Accordingly, the most common phylogenetic group was B2 (13/26, 50%), followed by phylogroup A (6/26, 23%), B1 (3/26, 11%), and C and D, with two isolates each. The epidemic strain ST131 O25b:H4-B2-*H*30-Rx represented 80% (8/10) of all ST131 studied during the periods 2011 and 2012 and 2016 and 2017 in Colombia (Table 2).

### 2.4. O25b-ST131 E. coli Clone Detection

In regards of the serotypes, all of the ST131 were 025:H4; ST44 were serotype 089:H4, and ST617 were serotype 089:H10. Of the two ST648, one had serotype O1:H6, and the other had an unknown O-group but belonged to H6.

### 2.5. Genes Related with Ciprofloxacin Resistance

A resistance to ciprofloxacin (CIP), found in 23/26 (88%) isolates, was attributed to mutations in the *gyrA* and *parC* (Table 2). Mutations at the quinolone resistance-determining regions (QRDR) of *gyrA* and *parC* (yielding to substitutions at Ser83 and Asp87 and Ser80 and Glu84 of the DNA gyrase and topoisomerase IV, respectively) were the most frequent. Of note, one ciprofloxacin-resistant isolate carried only the Asp87Tyr substitution in GyrA, lacking the Ser83Leu change that typically co-occurs. Likewise, only one isolate belonging to ST131 presented the Glu84 substitution. Lastly, a low prevalence of *qnr* genes (3/26, 11.5%) was found among these isolates.

### 2.6. Virulence Factor-Encoding Genes and Incompatibility Groups

Regarding the virulome, the most frequent virulence factor (VF)-encoding genes detected were *gad* (50%), *hlyD* (92%), *iutA* (88%), K1 and K2 (96% each), and *traT* (88%) (Figure S1). Other VF-encoding genes, such as *iha*, *sat*, and *pap* genes, were detected frequently among ST131 *E. coli* isolates, whereas the *pap(ACGH)* genes were associated with the *H*30-Rx subclone. Lastly, the most prevalent plasmid types found were of incompatibility groups IncFIA (20/26, 77%), IncFIB (24/26, 88%), and IncFII (23/26, 92%).

## 3. Discussion

ExPEC is one of the most important causes of BSIs and UTIs. Particular attention has been on the *E. coli* ST131 clone, which is considered a high-risk clone due to its epidemic potential, in both community and hospital settings, and its virulence and high level of antimicrobial resistance [10]. As expected, ST131 accounted for the majority (38%) of the STs detected in this study. Other ExPEC epidemic clones that have been detected among ESBL producers include ST405, ST38, ST648, ST410, and ST1193 [8], among which, ST405 and ST648 were also detected among the strains included.

According to the susceptibility testing, carbapenems were found to be the most active against the isolates included in this study, followed by fosfomycin. This result confirms that carbapenem resistance is still low among *E. coli* isolates in Colombia, given that only two strains, which were isolated in 2016, were resistant to these β-lactams. In accordance to the increasing reports of pathogenic isolates co-expressing two or more carbapenemases [11], one of the carbapenem-resistant ExPEC harbored both *bla*_KPC-2_ and *bla*_NDM-1_ genes. This gene combination has been reported worldwide and possesses a great challenge for therapeutic options [12].

Currently, the ESBL most widely distributed globally is CTX-M-15 [1]. In the ExPEC isolates included in our study, the acquisition of *bla*_CTX-M-15_ and *bla*_OXA-1_ were the most common β-lactam resistance-conferring mechanisms. We also noted that some of the isolates harboring the intact and wild type versions of *bla*_CTX-M-15_ were susceptible to ceftazidime, an uncommon phenotype that has been reported previously [13]. Based on what has been described for other antimicrobial resistance determinants [14], we asserted that the *bla*_CTX_M_ copy number and changes in the upstream genetic environment (e.g., promoter region) affect the expression levels of CTX-M. This, combined with changes in the outer membrane, could give rise to different phenotypes [15]. Additionally, 65% of *E. coli* strains carried the *aac(6′)Ib-cr* gene, which encodes for an aminoglycoside-modifying enzyme that provides resistance to amikacin and other aminoglycosides but is also known to confer low-level resistance to ciprofloxacin [16].

In general, we did not observe differences in the resistance to β-lactams, ciprofloxacin, and fosfomycin in ST131 compared to non-ST131 isolates. However, we found that the non-ST131 isolates carried more resistance genes to aminoglycosides, macrolides, trimethoprim, and sulfonamides (Table 1). An increased resistance to antibiotics in non-ST131 was already previously reported by Hojabri et al. [17].

*E. coli* ST131 strains are mostly of serotype O25:H4, with a specific O25b type. However, ST131 *E. coli* isolates of serotype O16:H5 have recently been identified in Europe, Japan, Australia, and Pakistan [18]. Additionally, the *H*30 subclone within *E. coli* ST131 has emerged as the dominant *E. coli* lineage among clinical isolates [19]. In our study, all of the ST131 clones had the *fimH* adhesin gene, and most of them were *H*30-Rx (8/10, 80%), as reported in previous studies [20]. The other two ST131 isolates belonged to the *H*35 subclone. All ST131 isolates carried both mutations described in *gyrA* and *gyrB.* Our results suggest that CIP resistance in *E. coli* ST131 in Colombia is associated with the lineages *H*30 and *H*35, which harbor a distinct *gyrA* and *parC* allele combination (*gyrA1AB*/*parC1aAB*). However, resistance to CIP was found in both ST131 and non-ST131 isolates (Table 2).

On the other hand, *E. coli* ST131 has an expansive range of VFs, which permits its survival in extraintestinal niches, including certain adhesins (*afa/dra* and *iha*); siderophore receptors (*iutA* and *fyuA*); toxins (*sat* and *hylA*); capsule variants (*kpsMT II* and *K2*); protection factors (*ompT*, *traT*, and *iss*); and others (usp and *malX*) [17]. In our study, the VFs that were almost exclusively associated with the *E. coli* O25:H4-B2-ST131 clone were *iha* and *pap(ACGH)*, which code for adherence factors, *senB*, which promotes toxin production, and *sat*, which encodes for a protease [19]. In addition, 66% of *E. coli* isolates, including all the ST131, have the *iss* gene, a virulence factor that promotes immune evasion by increasing serum survival [21]. Banerjee et al. [19] identified three virulence genes (*iha*, *sat*, and *iutA*), which are more prevalent among *H30* than in non-*H*30 ST131 isolates [19]. We found the *iha* and *sat* genes in both *H*30 and *H*35 ST131 *E. coli* subclones and differences in the *pap(ACGH)* genes, which were exclusively associated with the *H*30-Rx subclone. Interestingly, it has been reported that the presence of this VF is associated with the production of pyelonephritis and sepsis, because these isolates are easier to colonize and invade the upper urinary tract [22]. Of the three blood isolates, two belonged to the ST131 subclone *H*30 and had the same virulence genes as the urinary isolates of subclone *H*30 (Supplemental Figure S1).

CTX-M-15 has been frequently reported on plasmids of the IncF incompatibility group, which have the ability to acquire resistance genes and disseminate rapidly [23]. These plasmids might carry important VFs implicated in the spread of epidemic clone O25b:H4-ST131 [24].

Our study faced some limitations, such as the fact that the number of sequenced *E. coli* isolates was too small for a significant evaluation and that most isolates were from urine and community onset infections.

## 4. Materials and Methods

### 4.1. Bacterial Isolates

Two sets of isolates were included in this study. The first set was composed by 15 *E. coli* CTX-M group 1-positive isolates collected between 2011 and 2012 from community-acquired UTIs, as defined by the Center for Disease Control and Prevention (CDC). The second set included 8 oxyimino-cephalosporins-resistant *E. coli* isolates recovered from UTIs and 3 *E. coli* isolates recovered from bloodstream infections and collected from 2016 to 2017. Isolates from the second set were randomly selected and could be from community and hospital-acquired infections.

### 4.2. Susceptibility Testing

Minimum inhibitory concentrations (MICs) were determined by the broth microdilution method using customized Sensititer plates (Trek Diagnostic Systems, East Grinstead, West Sussex, UK) following the manufacturer’s recommendations. MIC values of ciprofloxacin (CIP) were determined with E-test™ (BioMerieux, Marcy L’Etoile, France). *E. coli* ATCC 25922 was used as the quality control strain, as per Clinical and Laboratory Standards Institute (CLSI) recommendations.

### 4.3. Whole-Genome Sequencing (WGS) and Analysis

Genomic DNA was extracted using the DNA Blood & Tissue Kit according to the manufacturer’s instructions (Qiagen, Hilden, Germany), followed by library preparation using the Nextera XT library (Illumina, San Diego, CA, USA), and sequenced on an Illumina MiSeq platform using the MiSeq v3 reagent kit (Illumina, USA). Phylogenetic groups (A, B1, B2, C, D, E, and F) were determined following the protocol published by Clermont et al. [25]. Sequence type (ST; according to the Achtman scheme [26]), O:H serotypes, serogroup, plasmid replicon types, virulence genes, clonotype determined by *fumC* and *fimH* (CH), and resistome were determined using the tools freely available at the Center for Genomic Epidemiology (http://www.genomicepidemiology.org/). The *H*30-Rx subclone was identified by detecting a specific single-nucleotide polymorphism (SNP) (G723A) from the allantoin permease (*ybbW*) gene [19]. The O25b subtype was determined by targeting 347 bp of the *para*(p)-aminobenzoate synthase (*pabB*) gene fragment. The *E. coli* K-12 strain was used as the reference genome for the WGS analysis (GenBank accession number NC000913).

Sequence data were deposited in the National Center for Biotechnology Information (NCBI) database (Bioproject: PRJNA608731).

### 4.4. Ethical Approval

The protocol was approved by the ethics committee of Centro Internacional de Entrenamiento e Investigaciones Médicas (CIDEIM) and the participating healthcare institutions (6 July 2011). Collection of the microbiological isolates was part of the regular diagnostic process, as established by each of the participating healthcare institutions. All subjects gave written informed consent in accordance with the Declaration of Helsinki.

## 5. Conclusions

Our study revealed the circulation of *E. coli* strains belonging to the C clade, O25b:H4-B2 ST131 clone, and *H*30-Rx subclone from outpatients and inpatients with UTIs and BSIs in Colombia similar to what has been reported on other continents, describing the spread of these endemic clones to this geographical region. To our knowledge, this is the first study that described the presence of the *H*30-Rx subclone in ST131 ExPEC in Colombia.

## Figures and Tables

**Table 1:** Demographic and microbiological data of CTX-M-harboring *Escherichia coli* isolates.

ID	City	Collection Date	Source	Ward	MLST	MIC (ug/mL)							
						CRO	CTX	CAZ	FEP	TZP	ETP	CIP	FOS
1EC	Bogotá	2011	Urine	ER	648	>4	>4	16	32	16/4	≤0.25	>4	≤32
2EC	Cali	2012	Urine	ER	44	>4	>4	32	16	16/4	≤0.25	>4	≤32
3EC	Cali	2012	Urine	ER	95	>4	>4	16	8	8/4	≤0.25	>4	≤32
4EC	Cali	2012	Urine	ER	131	>4	>4	16	8	4/4	≤0.25	4	≤32
5EC	Cali	2012	Urine	ER	131	>4	>4	8	16	≤2/4	≤0.25	>4	≤32
6EC	Cali	2012	Urine	ER	155	>4	>4	1	4	≤2/4	≤0.25	>4	>128
7EC	Cali	2012	Urine	ER	617	>4	>4	64	32	8/4	≤0.25	>4	≤32
8EC	Cali	2012	Urine	ER	617	>4	>4	8	16	8/4	≤0.25	>4	≤32
9EC	Cali	2012	Urine	ER	131	>4	>4	16	16	16/4	≤0.25	>4	128
10EC	Cali	2012	Urine	ER	44	>4	>4	64	32	16/4	≤0.25	>4	≤32
11EC	Cali	2012	Urine	ER	131	>4	>4	4	4	4/4	≤0.25	>4	≤32
12EC	Cali	2012	Urine	ER	405	>4	>4	32	16	8/4	≤0.25	>4	64
13EC	Cali	2012	Urine	ER	44	>4	>4	16	16	8/4	≤0.25	>4	≤32
14EC	Bogotá	2012	Urine	ER	131	>4	>4	16	32	8/4	≤0.25	>4	≤32
15EC	Bogotá	2012	Urine	ER	44	>4	>4	16	8	8/4	≤0.25	>4	≤32
16EC	Ibague	2017	Urine	ER	351	>4	>4	>32	64	32/4	≤0.25	0.5	≤32
17EC *	Cucuta	2017	Blood	ER	131	>4	>4	16	16	≤2/4	≤0.25	>4	≤32
18EC	Cali	2016	Urine	HOSP	648	>4	>4	>32	32	>128/4	>32	>4	≤32
19EC *	Barranquilla	2017	Blood	HOSP	405	>4	>4	16	32	8/4	≤0.25	>4	≤32
20EC	Pereira	2017	Urine	HOSP	131	>4	>4	32	32	8/4	≤0.25	>4	≤32
21EC	Pasto	2017	Urine	ER	126	>4	>4	16	8	≤2/4	≤0.25	4	≤32
22EC	Ibague	2017	Urine	ER	131	>4	>4	32	32	32/4	≤0.25	>4	≤32
23EC	Bucaramanga	2017	Urine	ER	212	>4	>4	>32	>64	≤2/4	8	≤0.25	64
24EC	Cucuta	2017	Urine	ICU	131	>4	>4	>32	32	>128/4	≤0.25	>4	≤32
25EC*	Pasto	2017	Blood	HOSP	131	>4	>4	16	8	4/4	≤0.25	>4	≤32
26EC	Ibague	2017	Urine	HOSP	SLV-ST 602	>4	4	8	8	≤2/4	≤0.25	≤0.25	64

CRO: ceftriaxone, CTX: cefotaxime, CAZ: ceftazidime, ER: emergency room, FEP: cefepime, HOSP: hospitalization, ICU: intensive care unit, MIC: minimum inhibitory concentration, MLST: Multilocus Sequence Typing, TZP: piperacillin/tazobactam, ETP: ertapenem, CIP: ciprofloxacin, and FOS: fosfomycin. (*) Isolates recovered from blood samples.

**Table 2:** Molecular data of CTX-M-harboring *E. coli* isolates.

ID	MLST	CTX-M	Phylogroup	Serogroup	*fimH*	Subclone	Allele gyrA/parC	CIP
4EC	131	CTX-M-15	B2	O25b:H4	30	*H*30-Rx	gyrA1AB/parC1aAB	4
5EC	131	CTX-M-15	B2	O25b:H4	30	*H*30-Rx	gyrA1AB/parC1aAB	>4
9EC	131	CTX-M-15	B2	O25b:H4	35	No-*H*30	gyrA1AB/parC1aAB	>4
11EC	131	CTX-M-15	B2	O25b:H4	30	*H*30-Rx	gyrA1AB/parC1aAB	>4
14EC	131	CTX-M-15	B2	O25b:H4	35	No-*H*30	gyrA1AB/parC1aAB	>4
17EC *	131	CTX-M-15	B2	O25b:H4	30	*H*30-Rx	gyrA1AB/parC1aAB	>4
20EC	131	CTX-M-15	B2	O25b:H4	30	*H*30-Rx	gyrA1AB/parC1aAB	>4
22EC	131	CTX-M-15	B2	O25b:H4	30	*H*30-Rx	gyrA1AB/parC1aAB	>4
24EC	131	CTX-M-15	B2	O25b:H4	30	*H*30-Rx	gyrA1AB/parC1aAB	>4
25EC *	131	CTX-M-15	B2	O25b:H4	30	*H*30-Rx	gyrA1AB/parC1aAB	>4
16EC	351	CTX-M-55	B1	O18a:H7	31		-	0.5
2EC	44	CTX-M-15	A	O89:H4	54		gyrA1AB	>4
10EC	44	CTX-M-15	A	O89:H4	54		gyrA1AB	>4
13EC	44	CTX-M-15	A	O89:H4	54		gyrA1AB	>4
15EC	44	CTX-M-15	A	O89:H4	54		gyrA1AB	>4
3EC	95	CTX-M-15	B2	O50:H4	27		gyrA1AB	>4
21EC ^a^	126	CTX-M-15	B2	H5	26		-	4
6EC	155	CTX-M-3	B1	O162:H19	32		gyrA1AB	>4
23EC	212	CTX-M-55	B2	O18ac:H49	38		-	≤0.25
12EC	405	CTX-M-15	D	O102:H6	27		gyrA1AB	>4
19EC *	405	CTX-M-15	D	O102:H6	27		gyrA1AB	>4
7EC	617	CTX-M-15	A	O89:H10	-		gyrA1AB	>4
8EC	617	CTX-M-15	A	O89:H10	-		gyrA1AB	>4
1EC	648	CTX-M-15	F	O1:H6	27		gyrA1AB	>4
18EC	648	CTX-M-15	F	H6	27		gyrA1AB	>4
26EC	*SLV*-ST 602	CTX-M-15	B1	H21	86		-	≤0.25

CIP: ciprofloxacin MIC, MLST: Multilocus Sequence Typing. (*) Blood stream isolates. ^a^ This isolate only harbors the known Asp87Tyr substitution in GyrA.

## Supplementary Materials

The following are available online at https://www.mdpi.com/2079-6382/9/12/899/s1: Figure S1: Virulence genotypes and incompatibility plasmids of the studied isolates.
