# Supplementary material for: Genomic Analysis of CTX-M-Group-1-Producing Extraintestinal Pathogenic E. coli (ExPEc) from Patients with Urinary Tract Infections (UTI) from Colombia
Source: Antibiotics (Basel). 2020 Dec 13;9(12):899. doi: 10.3390/antibiotics9120899 (PMC7763464; doi:10.3390/antibiotics9120899)
Supplement: Supplementary file 1 [file antibiotics-09-00899-s001.pdf]

antibiotics

MDPI

Article

Genomic Analysis of CTX-M-Group-1-Producing Extraintestinal Pathogenic *E. coli* (ExPEc) from Patients with Urinary Tract Infections (UTI) from Colombia

Elsa De La Cadena [1,*], María Fernanda Mojica [1,2,3], Nathaly Castillo [1], Adriana Correa [4,5], Tobias Manuel Appel [1], Juan Carlos García-Betancur [1], Christian José Pallares [1,6] and María Virginia Villegas [1,6]

1 Grupo de Investigación en Resistencia Antimicrobiana y Epidemiologia Hospitalaria, Universidad El Bosque, Bogotá 110121, Colombia; mfm72@case.edu (M.F.M.); ncastillop@unbosque.edu.co (N.C.); tobiasm.appel@gmail.com (T.M.A.); jgarciab@unbosque.edu.co (J.C.G.-B.); icako@hotmail.com (C.J.P.); mariavirginia.villegas@gmail.com (M.V.V.)

2 Department of Infectious Diseases, Case Western Reserve University, Cleveland 44106-7164, OH, USA

3 Research Service, Louis Stokes Veterans Affairs Medical Center, Cleveland, OH 44106-7164, USA

4 Centro Internacional de Entrenamiento e Investigaciones Médicas (CIDEIM), Cali 760031, Colombia; adriana.correa@imbanaco.com.co

5 Facultad de Ciencias Básicas, Universidad Santiago de Cali, Cali 760031, Colombia

6 Comité de Infecciones y Vigilancia Epidemiológica, Centro Médico Imbanaco, Cali 760031, Colombia

* Correspondence: ecadenav@unbosque.edu.co; Tel.: +57−1−6489-000

Received: 18 October 2020; Accepted: 27 November 2020; Published: date

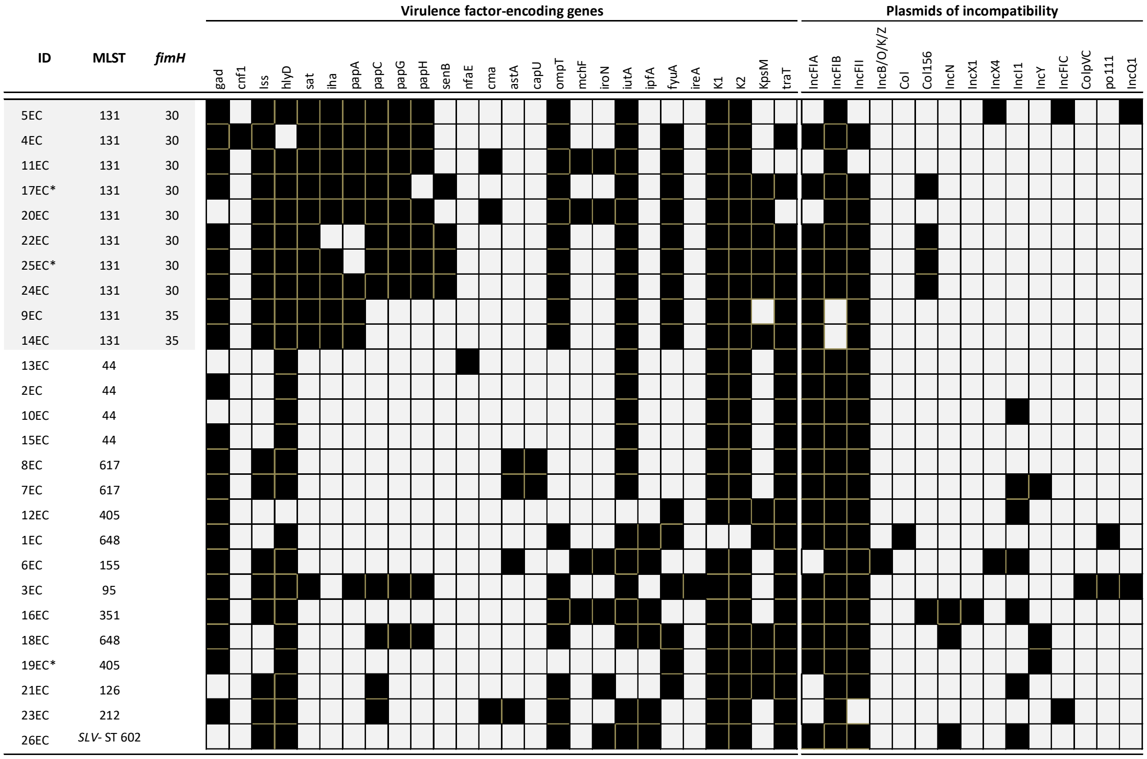

Figure S1. Virulence genotypes and incompatibility plasmids of the studied isolates. Black squares represent the presence of the respective virulence gene or incompatibility plasmid, and grey represents its absence. (*) Isolates recovered from blood samples.
